# C. Brandon Ogbunu(gafor)

**DOI:** 10.1093/gbe/evad056

**Published:** 2023-04-04

**Authors:** Adri K Grow

**Affiliations:** Department of Biological Sciences, Smith College, Northampton, MA 01063, USA

We continue our biography section, featuring Dr C. Brandon Ogbunu(gafor). The following is based on a December 2022 interview with Brandon.

## How Did You Become a Scientist?

Like in evolution, Brandon says becoming a scientist was due to a combination of forces that were small and seemingly random. From an early age, Brandon had a proclivity toward understanding the natural world, which came directly from his mother who was a school teacher. She had a love for science and more broadly a love of ideas, creativity, nature, and science fiction that provided an important framework for Brandon. She also had an enormous respect for researchers and scientists, embraced diverse perspectives, and questioned the natural world around her. Growing up in this environment, Brandon gravitated toward a future studying biology.

On a path to pursue biology, Brandon's childhood presented difficulties, including seeing the socioeconomic challenges his mother faced as a single parent of three in New York City. As a result of frequent moves, Brandon was not able to get into a rhythm at school and was never a particularly committed student despite his interests in reading and conceptualizing big ideas. Brandon finished high school as a mediocre student who had invested little effort, but transitioning to college sparked a desire to apply himself to follow his passion in the sciences. Howard University, a historically Black college and university, is where Brandon caught fire academically for the first time, learning how to navigate success in the classroom while gaining a group of close supportive friends.

After exploring majors such as computer science and math, Brandon decided to focus on chemistry after being inspired by the amazing set of instructors in the department. Brandon chose to further refine his area of study in biological chemistry, inspired by growing up in the 1990s in an urban setting during the ongoing HIV/AIDs epidemic, having always been curious about disease and microbes. Brandon was excited by the way scientists work to solve complicated problems, especially those with public health implications. These interests led him to study the herpes virus during an exchange at the University of California, Berkeley. Following this program, Brandon became an undergraduate researcher in Dr. Susan Gottesman's lab at the National Cancer Institute. Here, Brandon worked on bacterial genetics and discovered just how foundational biology would be to his future reasoning and developing career.

Following his undergraduate graduation, Brandon was a Fulbright Fellow in Kenya where he studied malaria. Though he had no prior experience in the field, he knew malaria was similar to HIV in that they were both infectious diseases with huge social consequences. From this point, choosing problems with a large societal component became a central part of Brandon's identity as a scientist. With these interests further developed, Brandon had a multitude of options to choose from as he thought about further education. He applied broadly and was admitted to medical and graduate school programs ranging from biophysics to bioengineering to combined degree programs. Brandon ultimately enrolled in an MD–PhD program at Yale University as it allowed for the most flexibility and exposure to diverse fields in science.

Starting his graduate program, Brandon immersed himself in the different scientific and medical environments offered and found that evolution was where he wanted to focus his energy. At the time, an evolutionary lens on medical problems was underappreciated and relatively new. Brandon joined Dr. Paul Turner's evolutionary virology lab at Yale where he studied issues such as disease emergence and drug resistance. Since that experience with Dr. Turner, Brandon never looked back. Following graduate school, Brandon focused on population genetics working as a postdoc with Dr. Daniel L. Hartl at Harvard University and the Broad Institute. Today, Brandon is an Assistant Professor in the Department of Ecology and Evolutionary Biology at Yale University where his lab aims to illuminate the ecological, evolutionary, and societal underpinnings of infectious disease.

**Figure:**
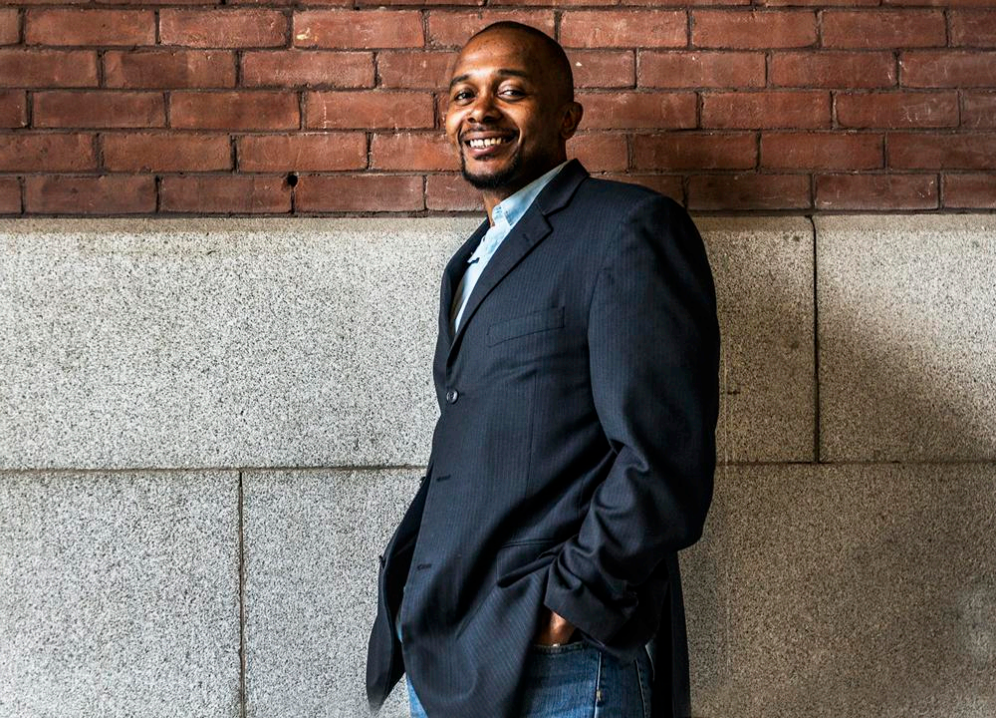
Dr. C. Brandon Ogbunu(gafor), Assistant Professor of Ecology and Evolutionary Biology at Yale University.

## Who Has Been Your Biggest Mentor or Influence on Your Career?

Brandon highlights two groups of people that had the biggest impact on him: those that had a very direct influence and those that had a symbolic influence. In the first category is the foundational start at home with his mother. Brandon states that he is a version of her but with more opportunities and that she's the reason for his good qualities scientifically. In the second category, since Brandon didn’t know any scientists or have any scientific role models, his heroes were hip-hop artists. Brandon found, and still believes, that hip-hop artists are some of the most expansive and creatively dynamic people he's ever come across and they are the people that taught Brandon through their music that anything is possible. Brandon applies the same mentality in his life, the notion that there are no limits, that creativity is essential, and that barriers can be broken down.

As Brandon moved on in his career, he met more people in the sciences that had a profound impact on him, one of them being Dr. Vernon R. Morris. Dr. Morris was Brandon's undergraduate research advisor, a young African American physical chemist who coauthored Brandon's first publication. Brandon fondly remembers Dr. Morris as a kind, brilliant, generous person who listened to the same music and talked the same way Brandon talked. Having this example of a scientist at the age of 18 for Brandon was immensely influential and had the biggest impact on his trajectory, so much so that Brandon aspired to be like him when he got older.

## What Are Some Challenges You’ve Faced in Your Career?

One of the main challenges Brandon actively overcomes in his career is not being tethered to the arbitrary rules and standards that are ingrained in the current academic system and especially in science. Brandon explains to students and colleagues that what makes science fun is creating your own set of rules to study the who, what, when, where, and why of the natural world, rather than fitting into the same mold that has become the expectation. It took Brandon some time to figure this out for himself early on, but today he prides himself on who he is and his research on actively avoiding such limitations to creativity and innovation.

## What Is Your Favorite Contribution to the Literature?

In this current stage of Brandon's career, his favorite thing to do is peer into the world to find places that offer unique perspectives and to leverage cultural relics to tell new stories. For example, Brandon does this often in his daily science; he’ll take a single data set and approach it from multiple different angles to say something interesting about how proteins evolve or how the mutation rate influences evolution, but he's even able to do this with books, film, and popular culture. This approach led to one of Brandon's favorite manuscripts coauthored by Michael D. Edge entitled “*Gattaca* as a lens on contemporary genetics: marking 25 years into the film's ‘not-too-distant’ future,” an article published in *Genetics* just in time for the 25th anniversary of the film's debut. Brandon was in his first year of college when the film was released, and 25 years later, he was able to mine it for technical ideas relevant to present-day genetic and genomic technology. Brandon's favorite aspect of this paper was the process of turning on the film, pressing pause, reading the literature, returning to the film, and watching the story unfold. For Brandon, this publication marked a triumph in his approach to science and in the space of creative collaboration where he thrives.

## What Do You Do for Fun Outside of Science?

Brandon's hobbies include reading fiction and comics, and watching films based in science, all things that have been historically labeled as “classic geek culture.” As a former boxer, Brandon also has a passion for sports, not just playing but also observing and studying them as well. Brandon enjoys engaging in the scholarly discourse of sports and even keeps up with the literature and writes about sports himself. Wherever he can, Brandon likes to expose himself to innovative and positive spaces, like the theater, for inspiration. In addition, Brandon is passionate about social issues and stays on top of these conversations through public speaking and social media. Lastly, one of Brandon's chief hobbies is writing, an unquestionably important piece of who he is.

## What's Some Advice for People Entering the Field of Science?

First and foremost, Brandon emphasizes that the rules in place within science and academia are simply goalposts and that they shouldn’t constrain your possibilities. Second, aside from peer review, grant proposals, and science engagement, Brandon doesn’t put effort into worrying about what other people think of him or the way he approaches his science. Brandon feels that as long as he is successful in publishing and writing grants, he has autonomy over the questions he pursues and the avenues in which those questions are answered. Brandon warns that unfortunately this internal voice of inspiration can get suppressed as early as high school and he encourages everyone to listen to that voice rather than work toward pleasing other people. He says that if this is possible, you’re able to dictate long-term happiness in your career.

